# Inter- and intra-reader agreement for gadoxetic acid–enhanced MRI parameter readings in patients with chronic liver diseases

**DOI:** 10.1007/s00330-019-06182-z

**Published:** 2019-04-18

**Authors:** Lucian Beer, Mattias Mandorfer, Nina Bastati, Sarah Poetter-Lang, Dietmar Tamandl, Dilyana Plamenova Stoyanova, Michael Christoph Elmer, Georg Semmler, Benedikt Simbrunner, Jacqueline C. Hodge, Claude B. Sirlin, Thomas Reiberger, Ahmed Ba-Ssalamah

**Affiliations:** 1 Department of Biomedical Imaging and Imaging-Guided Therapy, Medical University of Vienna, Waehringer Guertel 18-20, 1090 Vienna, Austria; 2 Division of Gastroenterology and Hepatology, Department of Medicine III, Medical University of Vienna, Vienna, Austria; 3 Vienna Hepatic Hemodynamic Laboratory, Medical University of Vienna, Vienna, Austria; 4 Liver Imaging Group, Department of Radiology, University of California, San Diego, La Jolla, CA 92093 USA

**Keywords:** Liver, Magnetic resonance imaging, Liver diseases, Gadoxetic acid, Liver function tests

## Abstract

**Objectives:**

To examine inter- and intra-observer agreement for four simple hepatobiliary phase (HBP)–based scores on gadoxetic acid (GA)–enhanced MRI and their correlation with liver function in patients with mixed chronic liver disease (CLD).

**Methods:**

This single-center, retrospective study included 287 patients (62% male, 38% female, mean age 53.5 ± 13.7 years) with mixed CLD (20.9% hepatitis C, 19.2% alcoholic liver disease, 8% hepatitis B) who underwent GA-enhanced MRI of the liver for clinical care between 2010 and 2015. Relative liver enhancement (RLE), contrast uptake index (CUI), hepatic uptake index (HUI), and liver-to-spleen contrast index (LSI) were calculated by two radiologists independently using unenhanced and GA-enhanced HPB (obtained 20 min after GA administration) images; 50 patients selected at random were reviewed twice by one reader to assess intra-observer reliability. Agreement was assessed by intraclass correlation coefficient (ICC). The albumin-bilirubin (ALBI) score, the model of end-stage liver disease (MELD), and the Child-Turcotte-Pugh (CTP) score were calculated as standards of reference for hepatic function.

**Results:**

Intra-observer ICCs ranged from 0.814 (0.668–0.896) for CUI to 0.969 (0.945–0.983) for RLE. Inter-observer ICCs ranged from 0.777 (0.605–0.874) for HUI to 0.979 (0.963–0.988) for RLE. All HBP-based scores correlated significantly (all *p* < 0.001) with the ALBI, MELD, and CTP scores and were able to discriminate patients with a MELD score ≥ 15 versus ≤ 14, with area under the curve values ranging from 0.760 for RLE to 0.782 for HUI.

**Conclusion:**

GA-enhanced, MRI-derived, HBP-based parameters showed excellent inter- and intra-observer agreement. All HBP-based parameters correlated with clinical and laboratory scores of hepatic dysfunction, with no significant differences between each other.

**Key Points:**

*• Radiological parameters that quantify the hepatic uptake of gadoxetic acid are highly reproducible.*

*• These parameters can be used interchangeably because they correlate with each other and with scores of hepatic dysfunction.*

*• Assessment of these parameters may be helpful in monitoring disease progression.*

**Electronic supplementary material:**

The online version of this article (10.1007/s00330-019-06182-z) contains supplementary material, which is available to authorized users.

## Introduction

Chronic liver diseases (CLD) are a major worldwide health problem. According to the Centers for Disease Control and Prevention, CLD were the 12th leading cause of death in the USA in 2015 [1]. Nonalcoholic fatty liver disease (NAFLD) [2, 3], alcoholic liver disease [4], and hepatitis C virus–induced liver disease [2] are the major etiologies of CLD in the USA and Europe, while hepatitis B virus is the leading cause in high-prevalence regions, such as Asia and Sub-Saharan Africa [5]. Although the prevalence of CLD from most etiologies has been stable, the prevalence of NAFLD has increased steadily, and this condition has now become the most common cause of CLD worldwide, affecting between 80 and 100 million individuals in the USA alone [2, 3].

Early diagnosis of CLD and accurate assessment of liver disease severity are key determinants for optimized patient management, since early treatment and lifestyle modification can arrest disease progression and even lead to improved hepatic function [6, 7] and a reversal of histological abnormalities [8, 9].

Assessment of liver function is an important issue for treatment individualization (etiological therapies and hepatocellular carcinoma) and follow-up, as well as for allocation of donors for living donor liver transplantation [10–12]. In daily practice, the severity of liver disease and liver function is often based on clinical signs of disease and biochemical blood parameters, such as the levels of albumin and bilirubin, as well as prothrombin time. Grading systems, such as the albumin-bilirubin ratio (ALBI) score, the Child-Turcotte-Pugh (CTP), or the model of end-stage liver disease (MELD), combine these parameters to determine liver function and are used for treatment decision-making. In addition to these tests, the indocyanine clearance, ^13^C methacetin breath test, and galactose elimination capacity are established methods for the evaluation of liver function [13].

In addition to these laboratory and clinical tests, magnetic resonance imaging (MRI) is commonly used in the diagnostic workup of patients with CLD [14, 15]. MRI elastography has shown promising results for the detection of fibrosis, especially in patients with NAFLD [16]. Therefore, it has recently been recommended by the American Association for the Study of Liver Diseases to be a clinically useful tool for the identification of advanced fibrosis in patients with NAFLD and, thus, might be used more commonly in the clinical routine in the future [17]. Diffusion-weighted MRI has been proposed in research studies as another method suitable for the detection of advanced fibrosis [18, 19]. However, the clinical value and applicability of this method are still under debate. Various attempts have also been made to use the hepatic uptake of gadoxetic acid (GA) as a noninvasive surrogate parameter for liver function. Recently, promising results have shown a high correlation between quantitative GA-derived hepatobiliary phase (HBP) scores with established parameters of liver function [15, 20–22]. Some of these methods often require specialized protocols, including dynamic contrast material enhancement analysis or T1 mapping at different time points [20, 22]. These acquisition protocols are often not easily integrated into clinical practice at most institutions. Besides these complex methods, several practical and simple quantitative imaging biomarkers of liver function have been introduced that require only two acquisitions (precontrast and 20-min HBP), which are routinely obtained for clinical care, and can be analyzed using simple equations [21, 23–25]. These include the relative liver enhancement (RLE), hepatic uptake index (HUI), contrast uptake index (CUI), and liver-to-spleen contrast index (LSI) [23, 25–28]. They all have been shown in individual studies to correlate with parameters of liver function. Importantly, there is currently no consensus as to which of these GA-MRI-derived scores is the most suitable for the assessment of hepatic function [29].

The aims of this study were (i) to assess the inter-observer agreement, (ii) to assess the intra-observer reliability for these four objective HBP imaging scores, and (iii) to correlate the four HBP imaging scores with established measurements of liver dysfunction, namely, the albumin-bilirubin (ALBI) score, the model of end-stage liver disease (MELD), and the Child-Turcotte-Pugh (CTP) score.

## Materials and methods

### Patients

For this retrospective study, our institutional ethics review board approved the data collection and analysis and waived the requirement for informed consent (Nr. 2027/2017). We searched our electronic medical record system for all patients with histopathologically or clinically proven CLD who underwent liver MRI with gadoxetic acid between January 2010 and December 2015. Inclusion criteria were (i) a gadoxetic acid–enhanced MRI with T1W imaging before and in the HBP 20 min after injection of the contrast agent, (ii) the presence of histopathologically or clinically confirmed CLD, and (iii) the availability of the following laboratory tests within 2 weeks of the MRI examination: albumin, bilirubin, creatinine, international normalized ratio (INR), and prothrombin time (PT). Exclusion criteria were previous or existing cancer of any organ system, large focal liver lesion(s) that would affect signal intensity (SI) measurements, biliary obstruction, and poor image quality including differences in MRI acquisition parameters between the precontrast and HBP images. All 287 patients (179 male, 108 female, mean age 53.5 ± 16.7 years, range 18–99) who met these criteria were included in the study (supplementary Figure 1).

### Clinical data

Demographic and clinical data were obtained from our institutional database. These included patient age, sex, cause of underlying liver disease, and—within 2 weeks before or after the MRI examination—measures of serum creatinine, INR, aspartate aminotransferase (AST), alanine aminotransferase (ALT), bilirubin, alkaline phosphatase, albumin, and cholinesterase, and MELD and CTP scores. The albumin-bilirubin ratio (ALBI) was calculated based on serum albumin and total bilirubin using the following formula: ALBI score = (log_10_ bilirubin [μmol/L] × 0.66) + (albumin [g/L] × − 0.085), while ALBI grade was defined by the resulting score (≤ − 2.60 = grade 1, greater than − 2.60 to ≤ − 1.39 = grade 2, greater than − 1.39 = grade 3). The ALBI score was chosen, as it is an objective score, solely based on serum albumin and total bilirubin, which correlates well with liver function/dysfunction [30]. The MELD natrium (Na) was also used in this study: MELD = 10 × (0.957 × ln (serum creatinine) + 0.378 × ln (total bilirubin) + 1.12 × ln (INR)) + 0.643, with a lower limit of 1 for all variables and with creatinine capped at 4. This was then applied to the MELD Na equation, MELD Na = MELD – SerumNa − (0.025 × MELD × (140 − SerumNa)) + 140, where sodium (Na) concentration is bound between 125 and 140 mmol/L. The MELD Na score was rounded to the nearest integer. Patients were subdivided into two groups based on their MELD score according to current recommendations for liver transplant listing [31]. Thus, there were 80 patients with a MELD score higher than or equal to 15, which represented patients with significantly impaired liver function, versus 206 patients with a MELD score lower than 15. Data are given in Table 1 and supplementary Table 1.

**Table 1:** Patients characteristics

Gender	
Male	179 (62.4%)
Female	108 (37.6%)
Age (years)	
Mean ± SD	53.5 ± 13.7
Range	18–99
Body weight (kg)	
Mean ± SD	79.7 ± 16.9
Range	43–136
Size (cm)	
Mean ± SD	173.3 ± 10.6
Range	135–196
Etiology of liver disease	
HCV	60 (20.9%)
Alcoholic liver disease	55 (19.2%)
HBV	23 (8.0%)
PSC	17 (5.9%)
PBC	9 (3.1%)
AIH	16 (5.6%)
CF	4 (1.4%)
NASH	15 (5.2%)
Miscellaneous/not specified	88 (30.7%)

*HCV*, hepatitis C virus; *HBV*, hepatitis B virus; *PSC*, primary biliary cirrhosis; *PBC*, primary biliary cirrhosis; *AIH*, autoimmune hepatitis; *CF*, cystic fibrosis; *NASH*, nonalcoholic steatohepatitis

### MRI protocol

MR examinations were performed at 3 T (Magnetom Trio, A Tim; Siemens Healthineers) using a combined, six-element, phased-array abdominal coil and a fixed spine coil. A standard dose of gadoxetic acid (0.025 mmol/kg; Primovist in Europe and Eovist in the USA; Bayer Healthcare) was injected intravenously at a rate of 1.0 mL/s, immediately followed by a 20-mL saline flush. The contrast-enhanced sequences comprised three-dimensional, T1-weighted, volume-interpolated, breath-hold examinations (VIBE) sequences. Axial dynamic images were acquired before and in the late arterial, portal venous (70 s), transitional (3 min), and hepatobiliary (20 min) phases after contrast injection. Arterial phase timing was determined using the bolus-tracking system. The MRI examination protocol also included axial in-phase and opposed-phase T1-weighted images, diffusion-weighted images (*b* values 50, 300, and 600 s/mm^2^), and conventional T2-weighted images. MR acquisition parameters are given in supplementary Table 2.

### Image analysis

**Fig. 1:**
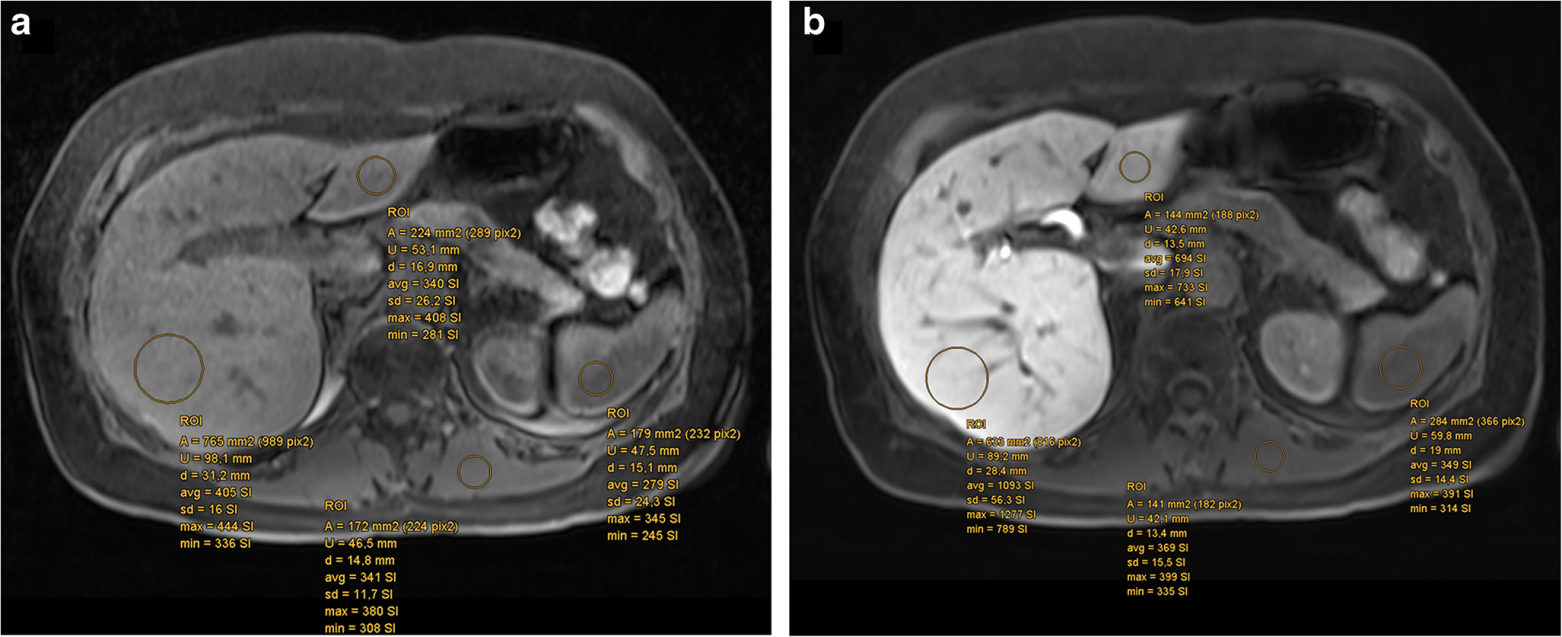
Axial MR shows placement of regions of interest for SI measurements of the liver parenchyma in the left and right lobes at different areas, as well as in the spleen and the left paraspinal muscle before (**a**) and 20 min after gadoxetic acid application (**b**). A, average; U, circumference; d, diameter; avg, average; sd, standard deviation; max, maximum; min, minimum; SI, signal intensity

Two radiologists, one board-certified with more than 20 years of experience (reader no. 1, A.B.) and the other in the fourth year of training (reader no. 2, L.B.), independently analyzed the axial unenhanced and HBP-enhanced 3D T1W images quantitatively on a picture archiving and communication system (PACS, workstation, Impax; Agfa) and performed volumetric analysis of the liver as described below. The readers were blinded to patient history and clinical data. One observer (no. 2, L.B.) repeated the measurements 4–10 weeks after the first session to assess intra-observer variability in 50 randomly assigned patients. The quantitative measurements came from four regions of interest (ROIs), which were circles chosen to be as large as possible, i.e., 2.0–5.0 cm^2^, within the liver, which included homogenous areas of the left lobe (segments II and III) and right lobe (segments VI and VIII). The mean value of these ROIs was calculated and used for further analysis. In addition, one ROI covering the maximum area of homogeneous tissue was placed in the spleen and left erector spinae, avoiding atrophic fatty areas, on the same slice as the liver ROIs (Fig. 1).

Quantitative image scores were calculated as previously described (Table 2) [23, 26]. Briefly, the RLE was calculated by subtracting the SI of the unenhanced images from the SI in the HBP, and dividing the difference by the SI of the unenhanced images. To calculate the CUI, the ratio of liver-to-paraspinal muscle SI, measured on the unenhanced and then on the enhanced images, was used. HUI was calculated by multiplying the liver volume (measured as described below) by the quotient of the SI of the enhanced liver and spleen. LSI was calculated by dividing the SI of the liver by that of the spleen on the enhanced images.

**Table 2:** Quantitative grading scores for gadoxetic acid uptake

RLE = (SI_Liver enh_ − SI_Liver unenh_) / (SI_Liver unenh_) × 100	
CUI = (SIR_enh_ / SIR_unenh_); SIR = (SI_Liver_ / SI_paraspinal muscle_)	
HUI = Volume_Liver_ (SI_Liver_ / SI_Spleen_ − 1)	
LSI = SI_Liver enh_ / SI_Spleen enh_	

*RLE*, relative liver enhancement; *CUI*, contrast uptake index; *HUI*, hepatic uptake index; *LSI*, liver-spleen index; *SI*, signal intensity; *SIR*, signal intensity ratio; *enh*, enhanced; *unenh*, unenhanced

Volumetry of the liver was performed using SyngoVia software (SyngoVia, Siemens Healthineers), with a semi-automatic workflow. The liver contour was manually delineated with the free-hand, volume-of-interest tool in the multimodal reading mode on multiple slices in either the axial or coronal plane, avoiding large vessels. After tracing the liver contour on adjacent images, the algorithm calculated the volume by interpolating between slices.

### Statistical analysis

Discrete variables were described by absolute numbers and percentages. Continuous variables were described by medians and interquartile ranges (Q1–Q3). Bland-Altman plots and the corresponding 95% limits of agreement were used to assess the agreement between the four image scores. Intra-observer intraclass correlation coefficients (ICCs) and their 95% confident intervals were calculated based on a single-measurement, absolute-agreement, two-way mixed-effects model. Inter-observer ICC variability and 95% confident intervals were calculated based on a single-rater, absolute-agreement, two-way random-effects model [32].

Associations between RLE, HUI, CUI, LSI, and clinical scores were investigated using Pearson’s correlation coefficient. The strength of correlation was categorized as very high (0.9–1.0), high (0.7–0.9), moderate (0.5–07), low (0.3–0.5), or negligible (0.0–0.3) [33]. An analysis of variance (ANOVA) with a Bonferroni post hoc analysis was used to compare the MRI-derived parameters with the ABLI score and CTP score. Receiver operating characteristic (ROC) curve analysis was performed to differentiate between patients with a MELD score higher than or equal to 15 and those with a MELD score below 15. The optimal cutoff values were estimated according to the Youden index. The areas under the curve (AUC) between the MRI-derived scores were compared using DeLong’s test and AUC, as well as classification rates, are reported. Data are given as median (interquartile range) or as box-plots, in which whiskers represent the 10th–90th quartiles. A two-sided *p* value of *p* < 0.05 was deemed statistically significant. Analysis was performed using SPSS, version 24 (IBM Corp).

## Results

There were 287 patients enrolled in this retrospective study. Patient characteristics are given in Table 1. Laboratory data are given in supplementary Table 1.

### GA-MRI scores

The median and IQR (Q1–Q3) were as follows: RLE 56.2 (39.8–97.6); CUI 1.37 (1.23–1.66); LSI 1.24 (1.08–1.68); and HUI 595 (199–1366) for observer no. 1 and RLE 56.7 (43.65–90.5); CUI 1.5 (1.45–1.56); LSI 1.25 (1.13–1.54); and HUI 629 (217–961) for observer no. 2.

### Intra-observer and inter-observer variability

**Fig. 2:**
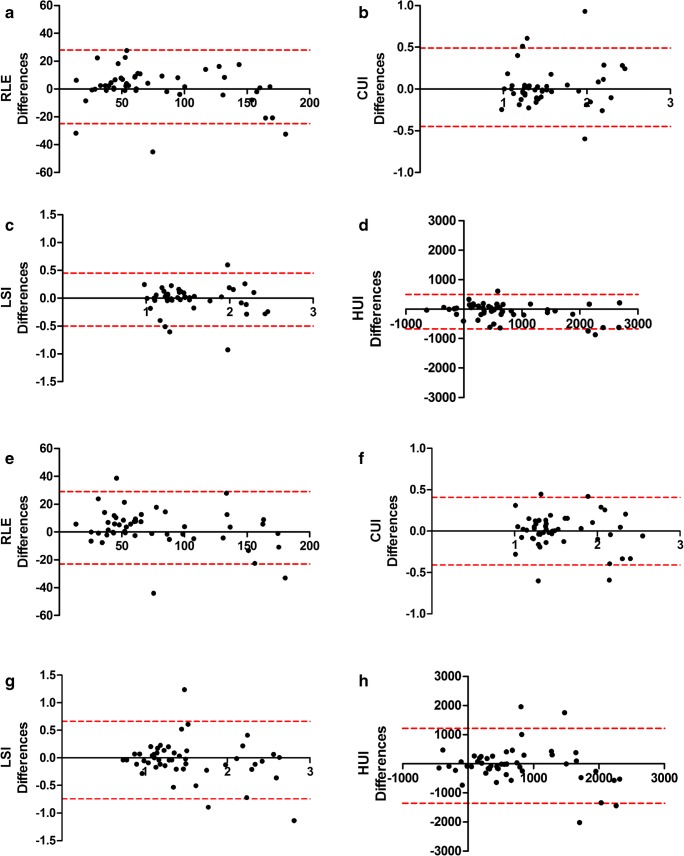
Intra-observer (panels **a**–**d**) and inter-observer (**e**, **f**) Bland-Altman plots were used to analyze the agreement between two evaluations of one observer or evaluations between observers. The difference between two evaluations was plotted on the vertical axis and the mean of the two evaluations was plotted on the horizontal axis. The solid (black) line represents the mean value for the data points and the dashed (red) line represents the 1.96 × SD. *n* = 50

The ICC and Bland-Altman analysis for inter- and intra-observer variability are summarized in Tables 3 and 4, respectively. Intra-observer ICCs ranged from 0.969 (0.945–0.983) for RLE to 0.814 (0.668–0.896) for CUI. Inter-observer ICCs ranged from 0.979 (0.963.0.988) for RLE to 0.777 (0.605–0.875) for HUI. Bland-Altman plots for inter- and intra-observer variability are shown in Fig. 2. There was no significant bias between observers for the calculation of RLE, LSI, and CUI, whereas there was a small bias between observers for HUI.

**Table 3:** Intra-observer agreement—interclass correlation coefficient, and Bland-Altman analysis

Intra-observer agreement		
	ICC	BA
RLE	0.969 (0.945 to 0.983)	1.6 ± 13.6 (− 25.2 to 28.4)
CEI	0.814 (0.668 to 0.896)	0.0 ± 0.2 (− 0.5 to 0.5)
LSI	0.961 (0.930 to 0.979)	0.0 ± 0.2 (− 0.5 to 0.5)
HUI	0.952 (0.914 to 0.973)	− 85 ± 296 (− 668 to 496)

*RLE*, relative liver enhancement; *CEI*, contrast enhancement index; *LSC*, liver-spleen index; *HUI*, hepatic uptake index

*ICC*, interclass correlation coefficient, based on single-measurement, absolute-agreement, 2-way mixed-effects model. Numbers in the parentheses are 95% confidence intervals

*BA*, Bland-Altman analysis. The mean difference, standard deviation, and, in parentheses, 95% limit of agreement are shown

**Table 4:** Inter-observer agreement—interclass correlation coefficient, and Bland-Altman analysis

Inter-observer agreement		
	ICC	BA
RLE	0.979 (0.963 to 0.988)	2.8 ± 13.6 (− 23.8 to 29.5)
CEI	0.899 (0.820 to 0.943)	0.0 ± 0.2 (− 0.4 to 0.4)
LSI	0.894 (0.812 to 0.940)	0.0 ± 0.4 (− 0.7 to 0.7)
HUI	0.777 (0.605 to 0.874)	− 45 ± 643 (− 1306 to 1216)

*RLE*, relative liver enhancement; *CEI*, contrast enhancement index; *LSC*, liver-spleen index; *HUI*, hepatic uptake index

*ICC*, interclass correlation coefficient, based on single-rater, absolute-agreement, 2-way random-effects model. Numbers in the parentheses are 95% confidence intervals

*BA*, Bland-Altman analysis. The mean difference, standard deviation, and, in parentheses, 95% limit of agreement are shown

### Correlation between four MR-derived HBP scores

**Fig. 3:**
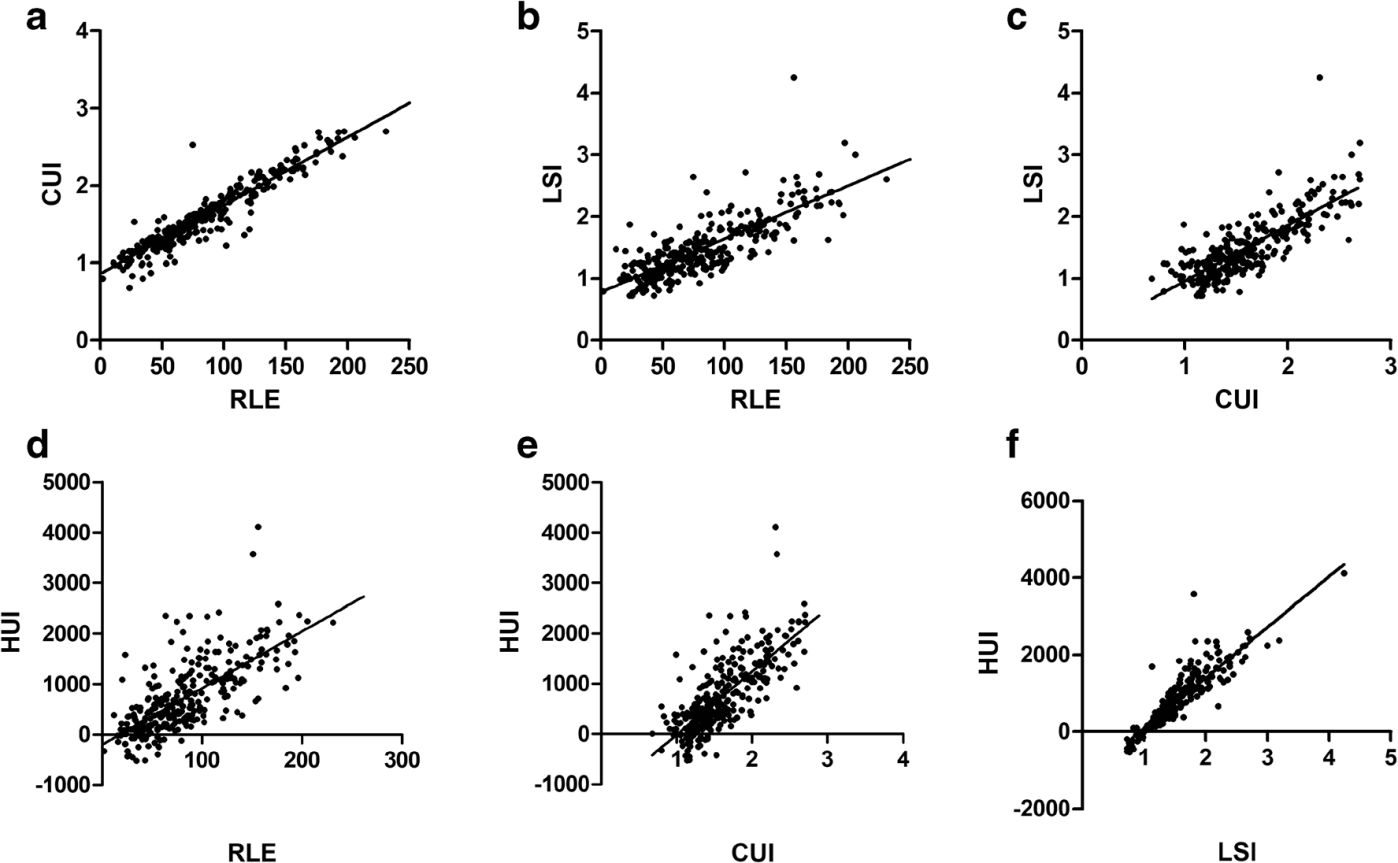
Correlation between the four MR-derived parameters: **a** CUI vs. RLE; **b** LSI vs. RLE; **c** LSI vs. CUI; **d** HUI vs. RLE; **e** HUI vs. CUI; and **f** HUI vs. LSI. RLE, relative liver enhancement; CUI, contrast uptake index; LSI, liver-spleen index; HUI, hepatic uptake index

As shown in Table 5 and Fig. 3a–f, there were strong positive correlations between all pairs of RLE, CUI, LSI, and HUI (*R* = 0.715–0.945, *p* < 0.001).

**Table 5:** Correlation between different quantitative MR parameters and laboratory data

		RLE	CUI	LSI	HUI	ALBI	MELD	CTP stage
RLE	*R*	1.0						
	*p* value							
CUI	*R*	0.945	1.0					
	*p* value	< 0.001						
LSI	*R*	0.792	0.802	1.0				
	*p* value	< 0.001	< 0.001					
HUI	*R*	0.715	0.747	0.906	1.0			
	*p* value	< 0.001	< 0.001	< 0.001				
ALBI	*R*	− 0.529	− 0.529	− 0.491	− 0.504	1.0		
	*p* value	< 0.001	< 0.001	< 0.001	< 0.001			
MELD	*R*	− 0.449	− 0.456	− 0.456	− 0.462	0.645	1.0	
	*p* value	< 0.001	< 0.001	< 0.001	< 0.001	< 0.001		
CTP stage	*R*	− 0.465	− 0.463	− 0.432	− 0.452	0.674	0.694	1.0
	*p* value	< 0.001	< 0.001	< 0.001	< 0.001	< 0.001	< 0.001	

*RLE*, relative liver enhancement; *CUI*, contrast uptake index; *LSI*, liver-spleen index; *HUI*, hepatic uptake index; *ALBI*, albumin-bilirubin grading system; *MELD*, model of end-stage liver disease; *CTP*, Child-Turcotte-Pugh

Associations between parameters were investigated using the Pearson’s correlation coefficient

### Correlation and discrimination between laboratory scores, clinical scores, and MR-derived HBP scores

**Fig. 4:**
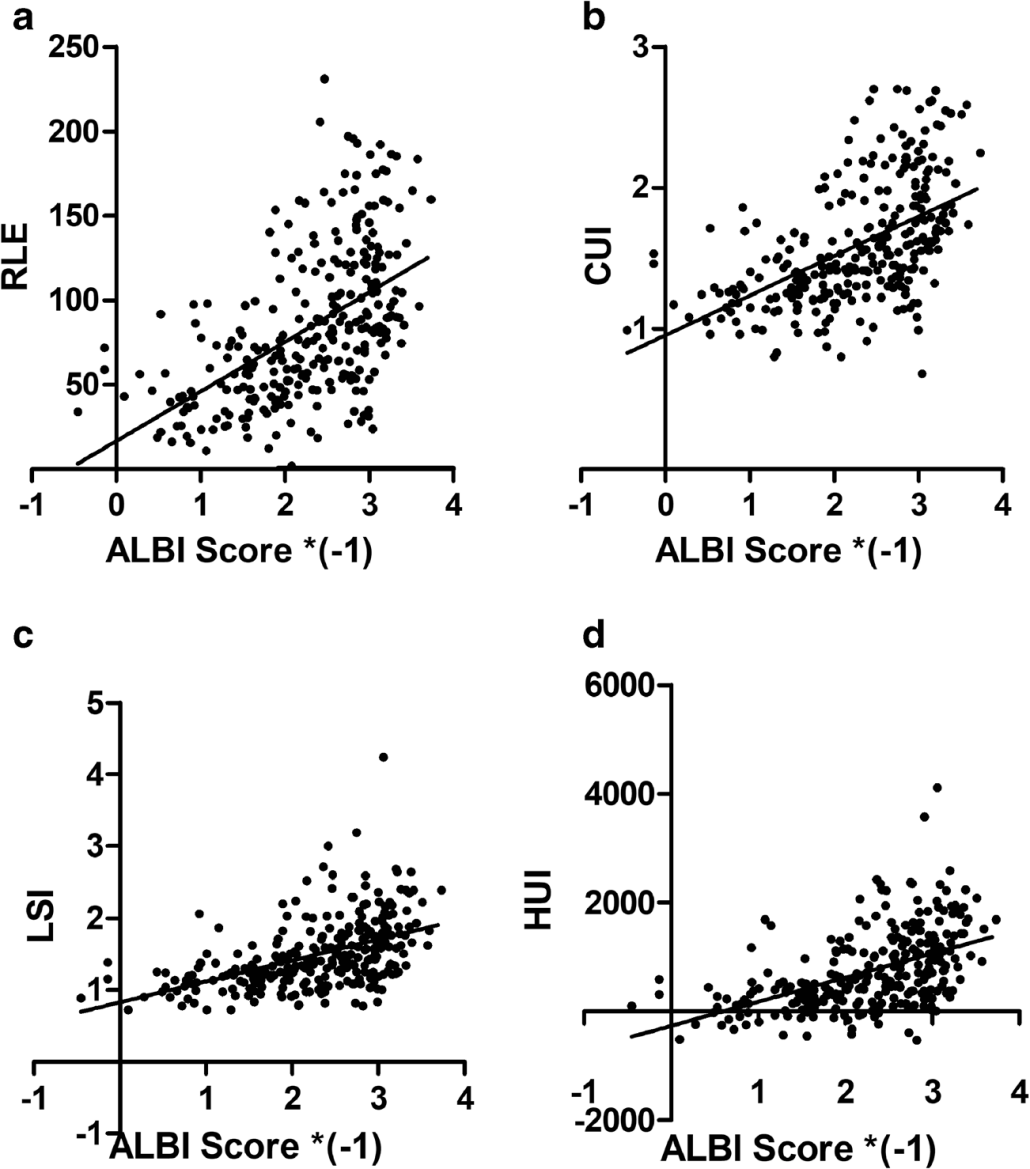
Correlation between the four MR-derived parameters and the ALBI score: **a** ALBI vs. RLE; **b** ALBI vs. CUI; **c** ALBI vs. LSI; **d** ALBI vs. HUI. *n* = 287. ALBI, albumin-bilirubin grading; RLE, relative liver enhancement; CUI, contrast uptake index; LSI, liver-spleen index; HUI, hepatic uptake index. ALBI Score *(-1), inverse ALBI score

There were negative correlations of moderate strength between each GA-MRI parameter and the ALBI score (RLE *R* = − 0.529; CUI *R* = − 0.529; LSI *R* = − 0.491; HUI *R* = − 0.504; *p* < 0.001) (Fig. 4, Table 5), as well as with the MELD score (RLE *R* = − 0.449; CUI *R* = − 0.456; LSI *R* = − 0.456; HUI *R* = − 0.462; *p* < 0.001) (supplementary Figure 2, Table 5) and the CTP score (RLE *R* = − 0.465; CUI *R* = − 0.463; LSI *R* = − 0.432; HUI *R* = − 0.452; *p* < 0.001) (Table 5).

**Fig. 5:**
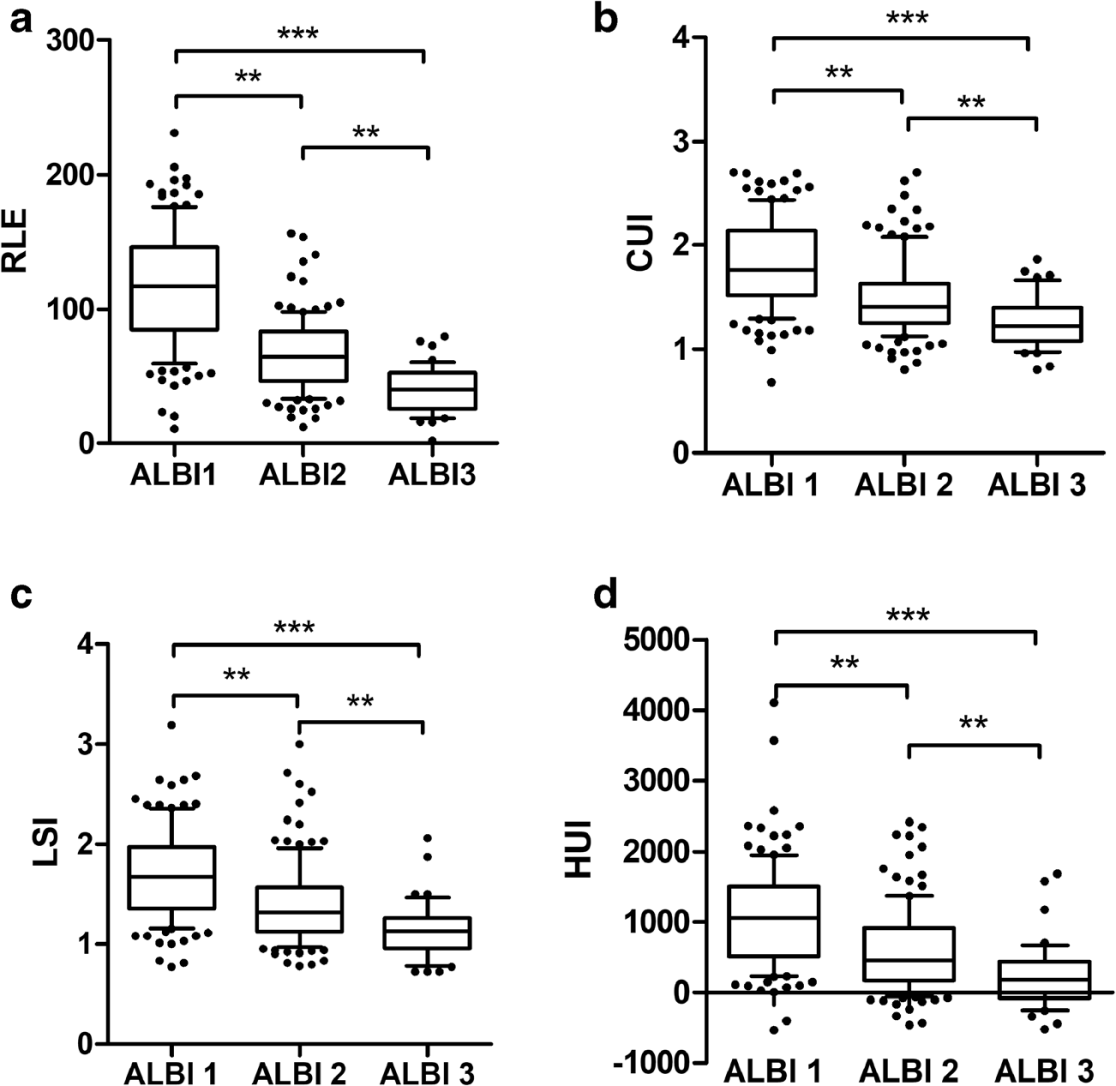
Results of **a** RLE, **b** CUI, **c** LSI, and **d** HUI for each ALBI grade category. The cutoff values were as follows: ≤ − 2.60 (grade 1); between − 2.60 and − 1.39 (grade 2); and ≥ − 1.39 (grade 3). **p < 0.01; ***p < 0.001 according to ANOVA with Bonferroni correction for multiple testing. Whiskers represent the 10th to the 90th percentiles; Black circle (●) denotes outliers; *n* = 287. ALBI, albumin-bilirubin grading; RLE, relative liver enhancement; CUI, contrast uptake index; LSI, liver-spleen index; HUI, hepatic uptake index

Patients with different ALBI grades and CTP scores had significantly different MRI-derived HBP scores (Fig. 5, supplementary Figure 2).

The optimal cutoff values for the four HBP scores, as well as their sensitivity, specificity, positive predictive value, and negative predictive value for the differentiation of the analyzed groups based on the MELD, are given in supplementary Table 3. There were no significant differences in the AUC (RLE 0.760, 95% confidence interval (CI) 0.699–0.821; CUI 0.757, 95% CI 0.697–0.817; LSI 0.778, 95% CI 0.718–0.838; HUI 0.782, 95% CI 0.723–0.840) between the groups (DeLong’s test, *p* > 0.05) (supplementary Figure 4).

## Discussion

Here, we show that the simple-to-obtain and simple-to-calculate MRI-derived HPB scores, i.e., the RLE, CUI, LSI, and HUI, have excellent intra- and inter-reader agreement. All HPB-derived scores showed a strong positive correlation with each other. Furthermore, all scores correlated moderately with liver disease severity, as assessed by the ALBI, MELD, and CTP scores, and, thus, show promise for accurately reflecting hepatic function in patients with CLD.

Over the past several years, several different quantitative methods for the measurement of hepatic uptake of GA have been introduced and have been shown to facilitate the noninvasive assessment of diffuse liver disorders. The most commonly used parameters are the RLE, CUI, LSI, and HUI, as they are solely based on changes of signal intensities in the HBP images compared with the unenhanced images, and therefore, easily obtainable. Various different groups have shown that these parameters correlate with established tests of liver function [21, 23–26, 28, 34–38]. Our study corroborates these results, highlighting that they all correlate with clinical parameters in a comparable strength, with no parameter being superior. Furthermore, we could show that all MRI parameters have a fair accuracy to differentiate between patients with significantly impaired liver function (MELD score ≥ 15) and patients with a MELD < 15. This threshold is of clinical importance, as a MELD score ≥ 15 is a criterion for liver transplantation listing, because the risk of dying from liver cirrhosis is greater than the postoperative mortality following liver transplantation [31]. What stands out when considering our results in detail is the relatively high positive predictive value that can be achieved by each of the MRI parameters, ranging from 0.876 for the RLE to 0.911 for the HUI. However, the negative predictive values are low, with ranges between 0.447 for the HUI and 0.532 for the RLE, indicating that these parameters are less suited to rule out liver dysfunction rather than to validate its presence.

As expected, all parameters showed an almost perfect positive correlation with each other and, importantly, were highly reproducible, as shown by their high intra- and inter-observer agreement.

With regard to the clinical applicability of the four scores evaluated, the RLE, the CUI, and the LSI can be easily obtained at each routine workstation, while the HUI calculation is more tedious and time consuming and currently cannot be performed as part of a routine MR examination. The next generation of MR scanners will likely come with post-processing software that will automatically measure the liver volume and will, therefore, help to evaluate the value of volume-based scores, i.e., functional volume for the assessment of liver function in CLD patients and/or in patients undergoing hepatectomy [12].

We decided to use the ALBI score as the reference standard in this study, as it is an objective and extensively validated indicator of hepatic function in different etiologies, stages of liver disease, and clinical scenarios [39, 40]. The ALBI score is predictive of survival even in the subgroup of patients with CLD who were classified as Child-Pugh A, and thus, it allows the subclassification of patients with less advanced CLD [30]. In contrast to the ALBI score, the MELD and CTP scores should be used only in patients with cirrhosis and not in CLD patients without cirrhosis. More specifically, the CTP score was initially developed to estimate the risk of mortality in patients who were undergoing surgery for variceal bleeding [41], while the MELD score was designed to estimate the mortality risk after transjugular intrahepatic portosystemic shunt [42], an intervention which is exclusively performed in decompensated advanced chronic liver disease/cirrhosis. Additional drawbacks of the MELD and CTP scores are well known. First, INR, which is included in the MELD score and in the CTP score, does not sufficiently reflect coagulopathy, and consequently, liver function, in patients with cirrhosis [43]. Second, two variables (i.e., hepatic encephalopathy and ascites) included in the CTP score are subjective. Finally, serum creatinine levels, as used in the MELD, may be altered by extrahepatic comorbidities. In contrast to the MELD and CTP scores, the ABLI score is more sensitive for patients with mild hepatic impairment and is not affected by kidney function and anticoagulation [44]. Therefore, we considered the ALBI score as the best clinical surrogate for hepatic function in our patient cohort. However, we also evaluated the MELD and CTP scores, as most previous studies that have evaluated the HPB-derived MRI parameters referred to these two scores. The MR parameters correlated well with the MELD and CTP scores as well.

The present study has some limitations. First, it is a retrospective data analysis, and thus, no sophisticated measures of hepatic function (e.g., indocyanine clearance or galactose elimination capacity) were available, and information about the ALBI score was not available on the date of the GA-enhanced MRI in any of our patients. However, the primary goal of this study was not to evaluate the correlation between GA-enhanced MRI parameters and liver function, as this topic has been extensively addressed in the literature, but, rather, to assess intra- and inter-reader agreement and comparability of different MRI-derived HBP scores. In addition, all patients had CLD, and thus, it is unlikely that relevant changes in liver function would have occurred in a so short period of time between the blood draw and GA-enhanced MRI. Second, we evaluated an inhomogeneous cohort that comprised patients with a host of CLD etiologies. However, the distribution of etiologies was a representative of the spectrum of CLD in the USA and Europe and, since our analysis was based on unselected real-life patients, this reflects the current referral practice at our center. Accordingly, our results are applicable in clinical practice. Moreover, the GA-enhanced MRI parameters correlated well with clinical scoring systems, i.e., ALBI, which is applicable for patients with CLD. Although histology is considered the gold standard for diagnosis, a clinical scoring system is a more practical comparator, as liver biopsy is not performed in the majority of CLD patients because it has many well-known limitations. ROI placement may cause some variation due to the possibly nonhomogeneous distribution of parenchymal changes. We, therefore, averaged the values from four ROI measurements across an area of the liver parenchyma to reduce sampling error. In addition, the ICC of a second reader demonstrated an excellent agreement for all HBP-derived parameters, indicating that these measurements provide robust results. Finally, the inclusion of parameters based on T1 relaxometry for the assessment of liver function would have improved the value of this manuscript.

In conclusion, MRI-derived HBP scores showed excellent inter- and intra-observer agreement and a moderate correlation with hepatic function. Thus, GA-enhanced MRI parameters have potential as excellent radiological tools for the evaluation of CLD patients in clinical practice. Accordingly, future studies that would evaluate the clinical value of GA-MRI-based indices, in combination with, or instead of, simpler blood tests, such as the ALBI, should use a simple, rather than a complex method, since all of these methods seem to provide the same information.

## Electronic supplementary material

ESM 1 (DOCX 636 kb)

## Abbreviations

ALBI: Albumin-bilirubin score
AUC: Area under the curve
CLD: Chronic liver disease
CTP: Child-Turcotte-Pugh
CUI: Contrast uptake index
GA: Gadoxetic acid
HBP: Hepatobiliary phase
HUI: Hepatic uptake index
ICC: Interclass correlation
INR: International normalized ratio
IQR: Interquartile range
LSI: Liver-spleen index
MELD: Model of end-stage liver disease
NAFLD: Nonalcoholic fatty liver disease
RLE: Relative liver enhancement
ROC: Receiver operating characteristic
ROI: Region of interest
