# Supplementary material for: Inter- and intra-reader agreement for gadoxetic acid–enhanced MRI parameter readings in patients with chronic liver diseases
Source: Eur Radiol. 2019 Apr 18;29(12):6600–10. doi: 10.1007/s00330-019-06182-z (PMC6828941; doi:10.1007/s00330-019-06182-z)
Supplement: Supplementary file 1 — (DOCX 636 kb) [file 330_2019_6182_MOESM1_ESM.docx]

**Supplemental Material**

| **sTable 1**. Laboratory Data | |
| --- | --- |
| Albumin, g x L^-1^  Median ± IQR (Q1-Q3) | 38.4 (32.6-42.6) |
| Alkaline phosphatase, U x L^-1^  Median ± IQR (Q1-Q3) | 104 (74-151) |
| Cholinesterase, U x L^-1^  Median ± IQR (Q1-Q3) | 5.0 (3.1-6.6) |
| ALT, U x L^-1^  Median ± IQR (Q1-Q3) | 33 (20-53) |
| AST, U x L^-1^  Median ± IQR (Q1-Q3) | 38 (29-64) |
| Gamma-GT, U x L^-1^  Median ± IQR (Q1-Q3) | 86 (41-178) |
| Direct Bilirubin, mg x dL^-1^  Median ± IQR (Q1-Q3) | 0.83 (0.37-2.59) |
| Indirect Bilirubin, mg x dL^-1^  Median ± IQR (Q1-Q3) | 0.79 (0.35-1.56) |
| Creatinine, mg x dL^-1^  Median ± IQR (Q1-Q3) | 0.84 (0.72-1.03) |
| INR  Median ± IQR (Q1-Q3) | 1.2 (1.1-1.4) |
| CTP  A  B  C | 169 (58.9%)  92 (31.1%)  26 (9.9%) |
| MELD  Median ± IQR (Q1-Q3) | 9 (6-15) |
| ALT, Alanin-Aminotransferase; AST, Aspartat-Aminotransferase; INR, international normalized ratio; MELD, model of end stage liver disease; CTP Child-Turcotte-Pugh; IQR (Q1-Q3) interquartile range | |

| **sTable 2: MRI parameter for 3T images** | | | | | | |
| --- | --- | --- | --- | --- | --- | --- |
| **Sequence** | **Section**  **Thickness**  **(mm)** | **TR**  **(msec)** | **TE**  **(msec)** | **FOV**  **(mm)** | **Phase Direction** | **Flip**  **Angle** |
| GRE-T1 (flash 2D) in-phase | 5 | 130 | 2.46 | 350 | AP | 70 |
| GRE-T1 (flash 2D) opposed-phase | 5 | 131 | 3.69 | 350 | AP | 70 |
| T1 VIBE SPAIR axial | 1.7 | 2.67 | 0.97 | 430 | AP | 13 |
| T1 VIBE SPAIR coronal | 2 | 2.6 | 0.92 | 500 | RL | 13 |
| T2 Haste coronal | 4.5 | 805 | 76 | 450 | RL | 141 |
| DWI axial TSE-EP | 6 | 1700 | 73 | 380 | AP | --- |
| T2 Haste axial fs | 5 | 1800 | 150 | 400 | AP | 150 |
| MRI, magnet resonance imaging, 2D = Two-dimensional, FOV = field of view, fs =fat saturation, GRE = gradient echo, SPAIR = spectral attenuated inversion recovery, TE = echo time, TR = repetition time, TSE = turbo spin echo; EP = echo-planar, VIBE = volumetric interpolated breath-hold examination. | | | | | | |

| **sTable 3.** Diagnostic performance to differentiate patient with different MELD scores | | | | | |
| --- | --- | --- | --- | --- | --- |
| **Score** | **Cut-Off** | **Sensitivity** | **Specificity** | **PPV** | **NPV** |
| **RLE** | 62.24 | 0.754 (0.689-0.811) | 0.725 (0.614-0.819) | 0.876 (0.810-0.909) | 0.532 (0.452-0.661) |
| **CUI** | 1.44 | 0.681 (0.613-0.744) | 0.750 (0.641-0.840) | 0.876 (0.807-0.906) | 0.476 (0.403-0.614) |
| **LSI** | 1.33 | 0.699 (0.631-0.761) | 0.759 (0.650-0.849) | 0.883 (0.817-0.912) | 0.492 (0.416-0.632) |
| **HUI** | 622.67 | 0.597 (0.527-0.665) | 0.848 (0.750-0.919) | 0.911 (0.846-0.932) | 0.447 (0.377-0.621) |
| RLE, relative liver enhancement; CUI, contrast uptake index; LSI, liver spleen index; HUI, hepatic uptake index; PPP, positive predictive value; NPV, negative predictive value; values in the brackets represent the lower and upper limit, respectively. | | | | | |

| **Supplementary Figure 1**  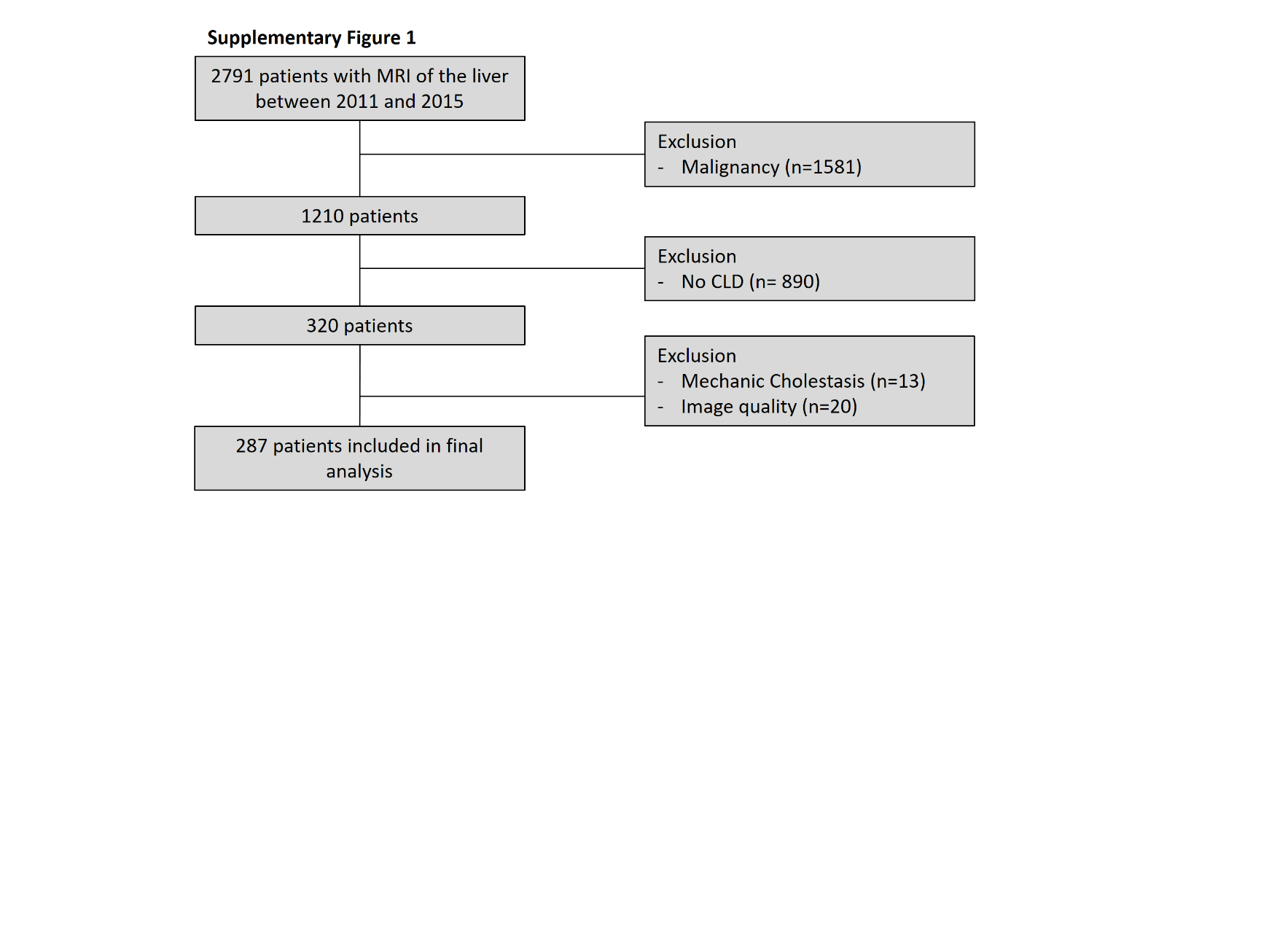 |
| --- |
| **sFigure 1.** Study flow chart |

| **Supplementary Figure 2.**  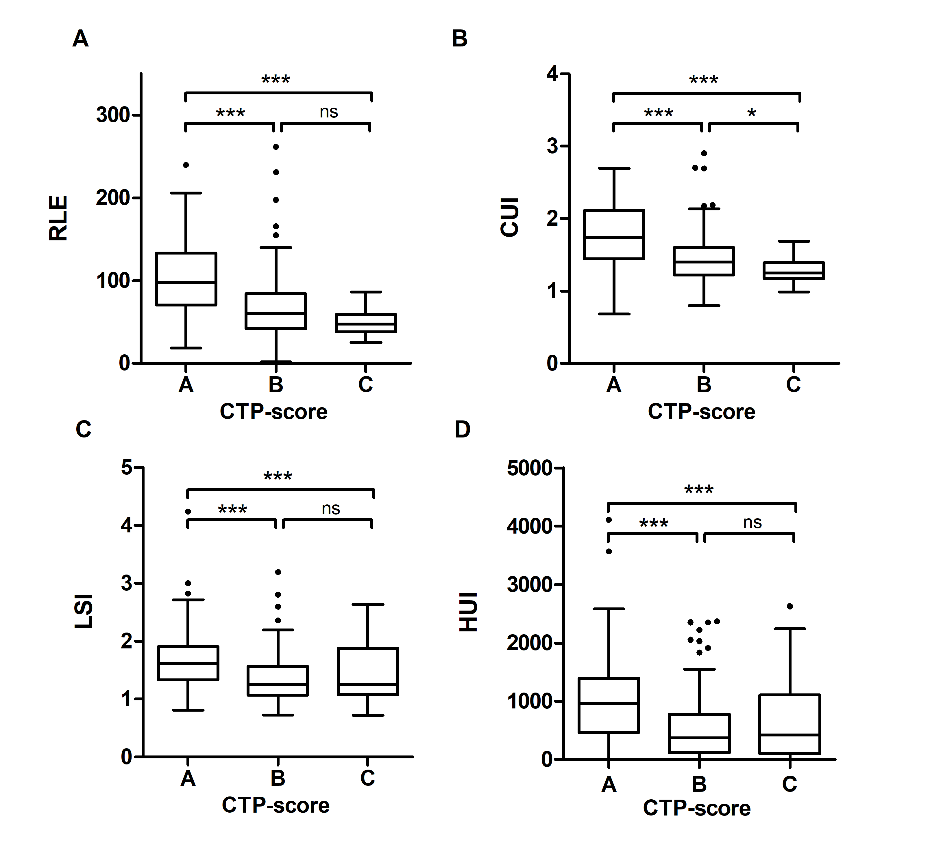 |
| --- |
| **sFigure 2.** Results of A) RLE, B) CUI, C) LSI, and D) HUI for each CTP score. * denotes p<0.05; ** denotes p < 0.01; *** denotes p < 0.001; ns, not significant according to ANOVA with Bonferroni correction for multiple testing. Whiskers represent the 10th to the 90th percentile; ● denotes outliers; n=287; CTP, Child-Turcotte-Pugh; RLE, relative liver enhancement; CUI, contrast uptake index; LSI, liver spleen index; HUI, hepatic uptake index. |

| **Supplementary Figure 3.**  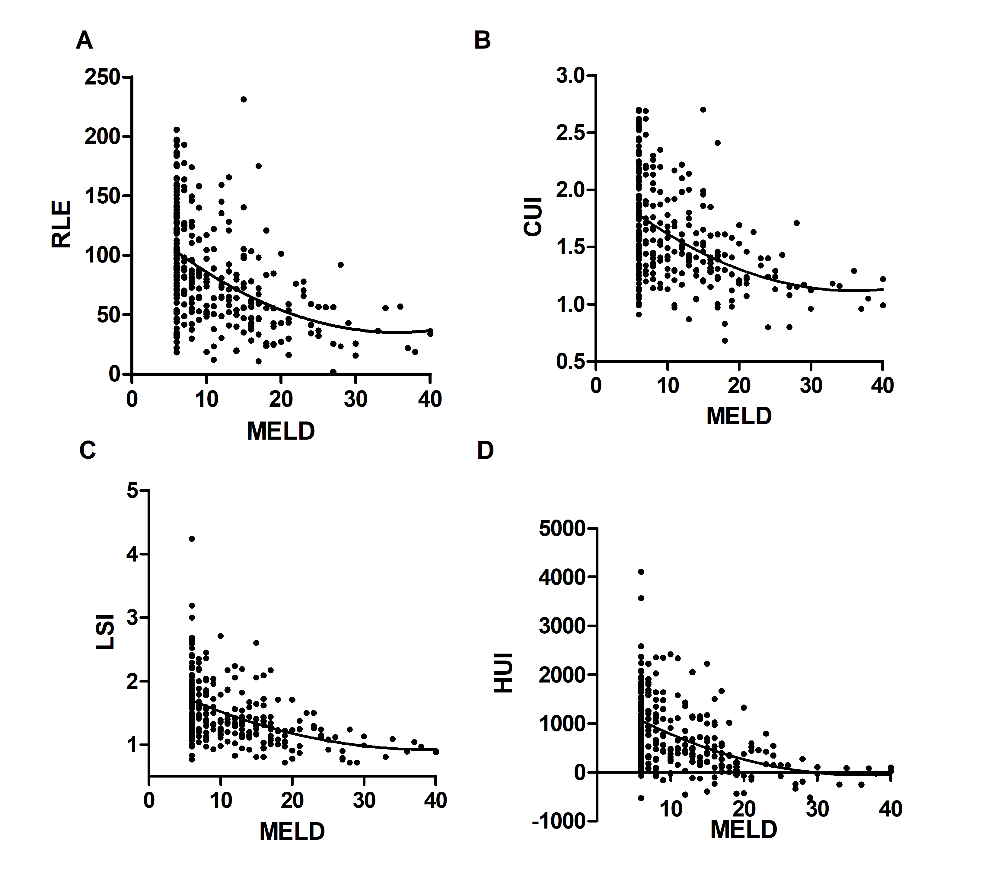 |
| --- |
| **sFigure 3.** Correlation between the four MR-derived parameters and the MELD score: A) MELD vs. RLE; B) MELD vs. CUI; C) MELD vs. LSI; D) MELD vs. HUI. n=287; MELD, model of end stage liver disease; RLE, relative liver enhancement; CUI, contrast uptake index; LSI, liver spleen index; HUI, hepatic uptake index. |

| **Supplementary Figure 4.**  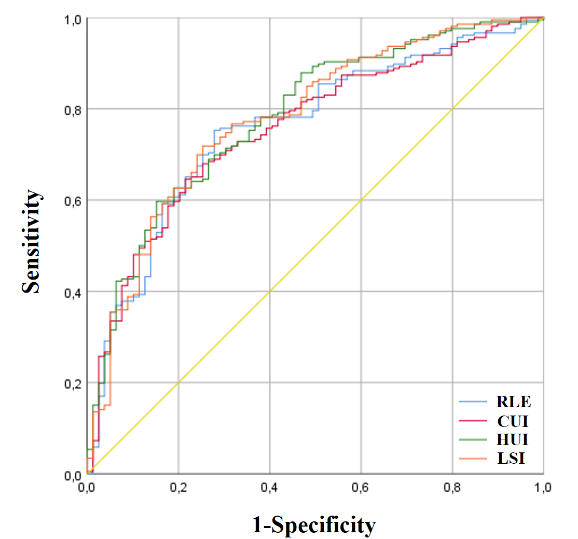 |
| --- |
| **sFigure 4.** Comparison of the relative liver enhancement (RLE), contrast uptake index (CUI), hepatic uptake index (HUI) and liver spleen index (LSI) in the detection of patients with a MELD-score equal or greater than 15. Area under the curve were comparable between the four scores ((RLE: 0.760 95% confidence interval [CI]: 0.699-8.21; CUI: 0.757, 95%CI: 0.697-8.17; LSI: 0.778, 95%CI: 0.718-0.838; HUI: 0.782, 95%CI: 0.723-0.840) |
